# Never mind the bug: no differences in infection-free survival after periprosthetic joint infections with *Staphylococcus aureus*, *Coagulase-negative Staphylococcus*, or *Streptococcus*

**DOI:** 10.3389/fmicb.2024.1503928

**Published:** 2025-01-03

**Authors:** Anders Brüggemann, Nils P. Hailer

**Affiliations:** Department of Surgical Sciences - Orthopedics, Uppsala University, Uppsala, Sweden

**Keywords:** PJI, *Streptococcus*, *Staphylococcus*, infection-free, survival

## Abstract

**Background:**

Periprosthetic joint infection (PJI) is a devastating complication following arthroplasty of the hip or knee joint and can be challenging to treat, depending on the underlying pathogen. There is still a debate whether streptococcal PJI are more difficult to treat than those caused by staphylococci. We aimed to investigate if the treatment results after PJI caused by *Staphylococci aureus* (*S. aureus*), *Coagulase-negative Staphylococci* spp. (*CoNS*) or *Streptococci* differ.

**Patients and methods:**

This study was designed as a retrospective observational study on patients with PJI caused by either streptococci or staphylococci in the hip or knee treated at a tertiary referral center between 1998 and 2021. Patients were identified in the local PJI register and data were collected by medical chart review performed minimum 1 year after the index PJI. Patients with polymicrobial infections or incomplete data were excluded, leaving 299 patients with streptococcal or staphylococcal PJI for final analysis. These patients were categorized according to the underlying pathogen: 114 were *S. aureus* 121 were *CoNS*, and 64 *Streptococci*. Infection-free survival was defined as the absence of (1) further surgery to the index joint due to PJI, (2) suppressive antibiotic therapy, and (3) death due to PJI and was assessed using the Kaplan–Meier method. Cox regression models were fitted to estimate the risk of infection relapse adjusted for relevant confounders.

**Results:**

We found no statistically or clinically significant difference in unadjusted survival between the three groups. Infection-free survival at 2 years was 71% (95%CI: 63–80) for *S. aureus*, 75% (95%CI: 67–84) for *CoNS*, and 60% (95%CI: 60–84) for *Streptococci*. The adjusted hazard ratios (HR) for the risk of infection relapse with *S. aureus* as the reference were 1.2 (95%CI: 0.7–2.0) for *CoNS* and 1.1 (95%CI: 0.6–2.0) for *Streptococci*. For all three groups of bacteria, survival was lower when DAIR was performed in comparison to exchange surgery.

**Discussion:**

In our cohort, there was no difference in infection-free survival between the three groups. Albeit limitations due to the study design, it seems that streptococcal PJI do not have to be considered more difficult to treat than their staphylococcal counterparts. Exchange surgery shows favorable results in all groups compared to DAIR.

## Introduction

Periprosthetic joint infection (PJI) occurs after 0.5–2% of total joint arthroplasties (Le Vavasseur and Zeller, 2022) but the incidence of PJI seems to be steadily increasing globally (Dale et al., 2023; Zeng et al., 2023; Karachalios and Komnos, 2021; Zardi and Franceschi, 2020), along with increasing antibiotic resistance (Benito et al., 2016). This is a relevant subject as a significant part of the population undergoes arthroplasty, while PJI and its insufficient treatment correlate with increased morbidity and mortality (Gerritsen et al., 2021). The most common causative pathogens in PJI are *Staphylococci* and *Streptococci* spp. (Triffault-Fillit et al., 2019; Zeller et al., 2018). The cornerstones of PJI are surgical intervention and long-term antibiotic treatment (McNally et al., 2021). Surgical strategies are divided into the implant-preserving combination of debridement, antibiotics, and implant retention (DAIR) and revision surgery, the latter performed either as a one- or a two-stage procedure (Zimmerli et al., 2004). The choice of surgical strategy depends on multiple factors such as duration of PJI symptoms and the time since previous surgery which both are indicative of how mature the biofilm formed on the implants is, and, therefore, how high chances are to cure the infection without extracting the implant (McNally et al., 2021). The affected patients’ medical comorbidities and the condition of the surrounding soft tissue are additional factors to weigh in (Osmon et al., 2013). Following surgery, streptococcal PJI is often treated by a single antibiotic, whereas staphylococcal PJI antibiotic therapy is mostly based on a combination of rifampicin, an antibiotic with anti-biofilm activity, with a second antibiotic such as clindamycin (Le Vavasseur and Zeller 2022). Streptococcal PJI is by some not considered a difficult-to-treat infection when compared to PJI caused by staphylococci, and according to one study, 89% of streptococcal PJI can be successfully treated, regardless of the applied surgical strategy (Lam et al., 2018). In contrast, several studies describe lower success rates after streptococcal PJI. Infection-free survival of only 57% is described in a study on 462 streptococcal PJI treated by DAIR (Lora-Tamayo et al., 2017), and only 60% successful outcomes after streptococcal PJI are found in a smaller cohort study on 30 patients treated with either DAIR or revision surgery (Akgün et al., 2017). In another cohort study, the overall infection-free survival rate of patients with streptococcal PJI is 71%, but decreases to 42% in the subgroup of patients treated by DAIR (Fiaux et al., 2016). These conflicting findings suggest a lack of knowledge regarding whether PJI caused by *Streptococci* should be considered a difficult-to treat infection and thereby potentially require a more extensive antibiotic or surgical treatment to increase infection-free survival than PJI caused by staphylococci. Staphylococci in their turn are heterogenic in their structure as well as bacterial virulence and hence present quite differently clinically. In general, the *CoNS*-caused PJI would present with milder symptoms, while PJI caused by *S. aureus* often are associated with pain, fever, local swelling and even sinus tracts when left untreated (Lourtet-Hascoët et al., 2016). However, *S. aureus* might be easier to treat in the absence of antibiotic resistance (Tornero et al., 2012).

Hence, there seems to be a knowledge gap: are the three bacteria similar to one another in terms of virulence and treatability? Or should one be considered more difficult-to-treat than the other?

## Patients and methods

This study was a retrospective observational study based on a cohort of PJI patients registered in the local PJI register at Uppsala University Hospital, a tertiary referral center. Patients with a PJI in the hip or knee treated from 1998 to 2021 who had a minimum follow-up of 1 year were included. The Swedish Ethical Review Authority granted ethical approval for retrospective analysis of PJI treated at Uppsala University Hospital from 1998 to 2021, registration number 2022–03466-02, including medical chart reviews.

### Participants/study subjects

**Figure 1:**
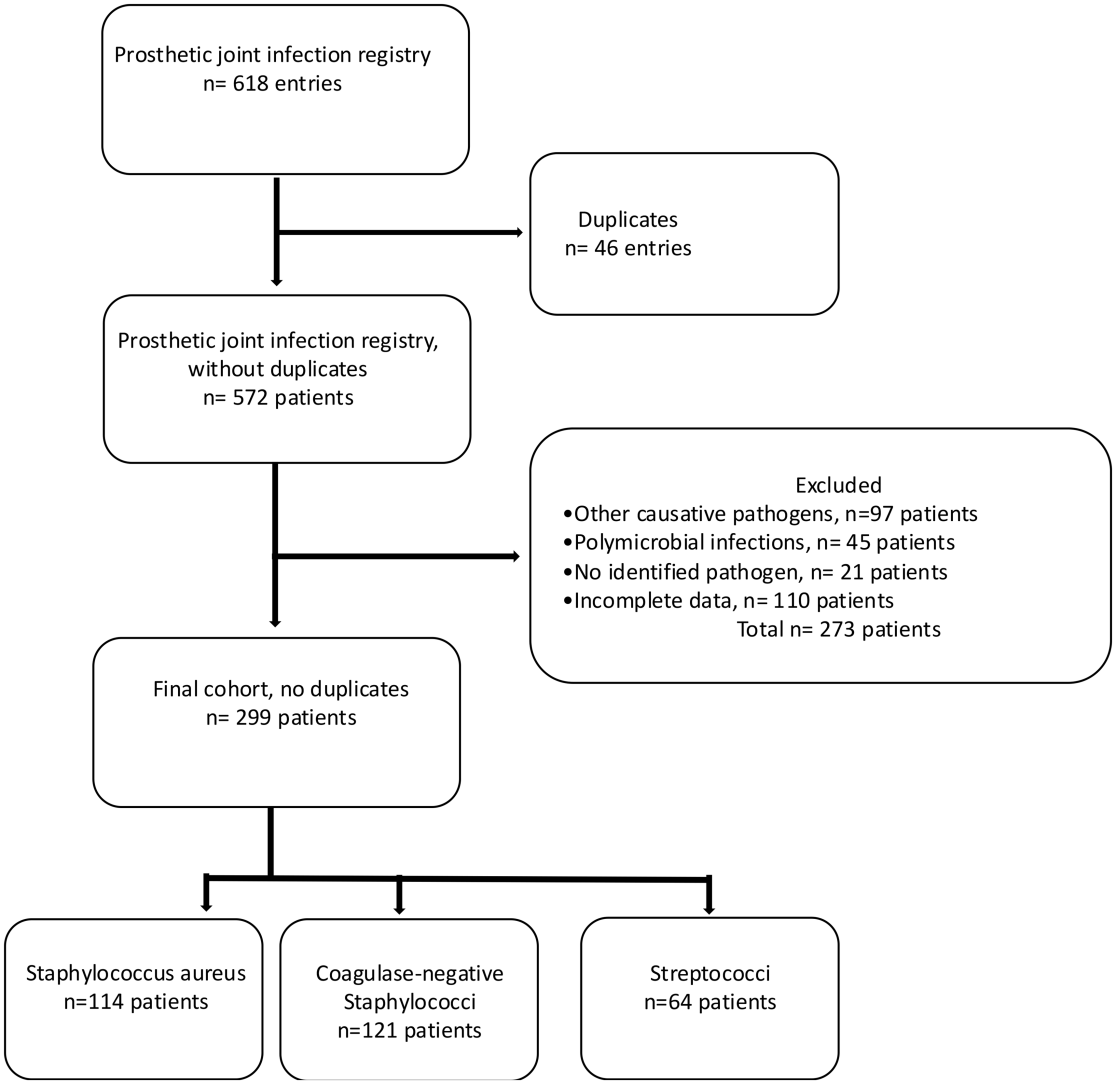
Flowchart depicting the inclusion of the final study cohort.

Patients with a surgically treated PJI in the hip or knee caused by streptococci or staphylococci were included, whereas patients with PJI caused by other pathogens than these two strains, those with polymicrobial infections, patients with a PJI caused by unidentified pathogens and those with incomplete data were excluded (Figure 1). In case one patient was found to have several infections only the chronological first one was included in this study in order to avoid dependency issues. The criteria for diagnosing PJI have changed over the study time, and for this study, the revised definition of PJI was used, i.e., a combination of clinical signs, blood and synovial fluid biomarkers, synovial fluid cytology, microbiology, histology, and nuclear imaging (Shohat et al., 2019). The presence of a joint communicating sinus tract, more than two samples of congruent intraoperative microbiological findings, more than 80% polymorphonuclear cells and elevated leukocyte cell count in synovial fluid are examples of findings where PJI was considered confirmed (McNally et al., 2021). Medical chart review was performed to assess findings of pre-, intra-, and postoperative cultures in tissue, synovial fluid, abscess drainage, blood, and wound secretion. The following hierarchy regarding the significance of culture findings was applied: (1) tissue, (2) synovial fluid, (3) abscess drainage, (4) blood, and (5) wound secretion, whereby tissue cultures were deemed most and cultures from wound secretion least representative. When multiple cultures were sampled the location with the highest significance rank was considered representative. If preoperative cultures indicated growth of more than one type of bacteria but intraoperative cultures showed only one, the divergent preoperative finding was considered a contamination, and patients were included, given otherwise fulfilled inclusion criteria. The included patients were categorized into three groups according to the findings: PJI caused by *S. aureus*, *CoNS*, or *Streptococci*.

Patients with PJI underwent either DAIR, one- or two-stage revision followed by antibiotic therapy at Uppsala University Hospital. Even though surgical strategies have changed over the years, some basic principles were consistent over the study period: Regardless the type of surgery, the scar tissue as well as any potential fistula is excised and a minimum of 5 tissue samples taken at an early stage during the procedure are collected and sent for analysis.

Excessive debridement is performed to remove all apparently infected tissue around the joint. In cases of DAIR, all modular parts are removed and sent for sonication (during the latest part of the study period). In case of 1- or 2-stage revision surgeries, all implants including all cement are carefully removed and the extracted implants are sent for sonication (during the latest part of the study period). Pulsed lavage of 6 liters 0.9% saline followed by soaking the wound with Prontosan^®^ (B. Braun, Germany) is performed before the wound is temporarily closed. After re-draping, the second stage advances making use of new, sterile instruments. Repeated pulsatile lavage with a minimum of 3 liters 0.9% saline and – if necessary – further debridement is carried out. Depending on the surgical strategy, new modular components (DAIR), new definite implants (1-stage) or a spacer (2-stage) are implanted followed by thorough closure of the wound.

Index surgery was defined as the first surgery where a monomicrobial PJI caused by *S. aureus*, *CoNS*, or *Streptococci* was treated at Uppsala University Hospital. Previous surgery was defined as the last surgery prior to index surgery. Previous and consecutive surgeries in the affected joint were examined. Information on pre-, intra-, and postoperative antibiotic treatment that had been administered was collected, including types of antibiotics, routes of administration, cause of altered antibiotic therapy and total duration of the antibiotic therapy.

Infection-free survival was the primary outcome and was defined as the absence of all of the following: renewed surgery of the index joint due to PJI, initiation of antibiotic suppression therapy, amputation, or death due to PJI, all due to the same bacteria as found at index surgery. Competing events were defined as re-revision surgery of the index joint, either due to infection caused by other pathogens or due to other reasons than infection, whereas non-informative censoring was defined as emigration (loss to follow-up), end of follow-up, or death due to other reasons than PJI, whichever came first. Outcome events were defined in time by the date of surgery when either a new pathogen or a relapse of the initial pathogen was identified by microbiological cultures, the date of initiation of antibiotic suppression therapy, the date of amputation, the date of death, the date of end of follow-up or the last medical chart entry, whichever came first. To minimize different sources of bias such as unmeasured confounders, selection bias or misclassification, thorough chart reviews were performed to minimize the effect on the mentioned sources of errors on the estimates.

Quantitative variable distributions were described using medians, and interquartile ranges, and categorical variables were summarized in frequency tables. The probability of infection-free survival (equal to the absence of relapse of infection, see previous definition) after index surgery was estimated with 95% confidence intervals (CI) using the Kaplan–Meier method, and Mantel–Haenszel’s log-rank test was applied to investigate differences between groups. Cox regression models were fitted to estimate the adjusted risk of infection relapse, expressed as a hazard ratio (HR) with 95% CI for patients with PJI caused by *CoNS* or *Streptococci* compared to those with PJI caused by *S. aureus*, adjusted for the confounders affected joint, age, gender, type of index surgery, i.e., DAIR, one-stage or two-stage exchange, the onset of the PJI, characterized as early, delayed or late as well as the type of surgery performed prior to the index surgery, i.e., primary arthroplasty, revision arthroplasty due to infection or revision arthroplasty due to any other reason than infection. Schoenfeld-residuals were plotted to confirm that the underlying assumptions of proportionality were met. Furthermore, one analysis stratified for the type of index surgery and one stratified for the type of infection, i.e., “early,” “delayed” or “late,” were performed to estimate differences in unadjusted survival between the three groups. The threshold for statistical significance was defined as *p* < 0.05.

The original register consisted of 572 unique patients and 46 entries representing patients who had PJI present in two or more joints, resulting in a total of 618 entries unique for one joint. After exclusion according to the previously stated criteria, a study cohort of 299 patients was identified; 114 with *S. aureus*, 121 with *CoNS*, and 64 with *Streptococci* (Figure 1). Date of index surgery varied from 2001 to 2021. Median follow-up time was 4.0 years (IQR = 1.0–7.7 years). More men (54.8%, *n* = 164) than women were identified, and more hips (63.5%, *n* = 190) than knees were affected. Median age of the patients was 72 years (IQR = 14, Table 1).

**Table 1:** Description of the study population.

	*CoNS* (*N* = 121)	*S. aureus* (*N* = 114)	*Streptococci* (*N* = 64)	Overall (*N* = 299)
Sex				
Women	54 (44.6%)	48 (42.1%)	33 (51.6%)	135 (45.2%)
Men	67 (55.4%)	66 (57.9%)	31 (48.4%)	164 (54.8%)
Joint				
Hip	83 (68.6%)	68 (59.6%)	39 (60.9%)	190 (63.5%)
Knee	38 (31.4%)	46 (40.4%)	25 (39.1%)	109 (36.5%)
Age at index surgery				
Median (IQR)	71 (14)	72 (16)	72 (11)	72 (14)
Previous surgery				
Primary arthroplasty	77 (63.6%)	84 (73.7%)	54 (84.4%)	215 (71.9%)
Revision surgery due to infection	10 (8.3%)	8 (7.0%)	1 (1.6%)	19 (6.4%)
Revision surgery for reasons other than infection	34 (28.1%)	22 (19.3%)	9 (14.1%)	65 (21.7%)
Onset of PJI				
Early	64 (52.9%)	61 (53.5%)	17 (26.6%)	142 (47.5%)
Delayed	35 (28.9%)	21 (18.4%)	19 (29.7%)	75 (25.1%)
Late	22 (18.2%)	32 (28.1%)	25 (39.1%)	79 (26.4%)
Missing*	0 (0%)	0 (0%)	3 (4.7%)	3 (1.0%)

*CoNS*, *Coagulase-negative staphylococci*; *S. aureus*, *Staphylococcus aureus*; IQR, inter quartile range; PJI, periprosthetic joint infection. *No information could be found on the timepoint of the surgery prior to the index surgery.

The most frequently performed surgery was DAIR, representing 74.9% (*n* = 224) of all index surgeries (Table 2), yet it was the chosen method in more cases among the PJI caused by *S. aureus* and *Streptococci* than in *CoNS*-caused PJI. Consequently, 2-stage revision surgery was more often performed in PJI caused by *CoNS*. A summary of antibiotic therapies is provided in Table 2 and intraoperative microbial findings are listed in Appendix 1.

**Table 2:** Details on given treatment.

	*CoNS* (*N* = 121)	*S. aureus* (*N* = 114)	*Streptococci* (*N* = 64)	Overall (*N* = 299)
Type of surgery				
DAIR	74 (61.2%)	99 (86.8%)	51 (79.7%)	224 (74.9%)
One-stage revision	10 (8.3%)	5 (4.4%)	6 (9.4%)	21 (7.0%)
Two-stage Revision	37 (30.6%)	10 (8.8%)	7 (10.9%)	54 (18.1%)
Route of administration, primary antibiotic^a^				
Intravenous	114 (94.2%)	108 (94.7%)	64 (100%)	286 (95.7%)
Oral	7 (5.8%)	5 (4.4%)	0 (0%)	12 (4.0%)
Missing	0 (0%)	1 (0.9%)	0 (0%)	1 (0.3%)
Days of antibiotic therapy, primary antibiotic				
Median (IQR)	7.0 (7.0)	6.0 (7.5)	5.0 (6.0)	6.0 (7.3)
Missing	3 (2.5%)	3 (2.6%)	1 (1.6%)	7 (2.3%)
Route of administration, secondary antibiotic^b^				
Intravenous	61 (50.4%)	55 (48.2%)	37 (57.8%)	153 (51.2%)
Oral	38 (31.4%)	51 (44.7%)	24 (37.5%)	113 (37.8%)
Missing	22 (18.2%)	8 (7.0%)	3 (4.7%)	33 (11.0%)
Days of antibiotic therapy, secondary antibiotic				
Median (IQR)	11 (28)	12 (30)	8.5 (37)	11 (29)
Missing	21 (17.4%)	12 (10.5%)	4 (6.3%)	37 (12.4%)
Total duration of antibiotic therapy				
Median (IQR)	64 (63)	92 (110)	99 (50)	89 (85)
Rifampicin therapy^c^				
No	56 (46.3%)	23 (20.2%)	56 (87.5%)	135 (45.2%)
Yes	65 (53.7%)	91 (79.8%)	8 (12.5%)	164 (54.8%)

*CoNS, Coagulase-negative Staphylococci; S. aureus, Staphylococci aureus; DAIR, debridement, antibiotics and implant retention; IQR, inter quartile range.*

a Defined as initially prescribed antibiotic regimen, including one or more drugs.

b Defined as secondary prescribed antibiotic regimen, including one or more drugs, after the initial regimen.

c Defined as registered Rifampicin therapy after index surgery.

## Results

**Figure 2:**
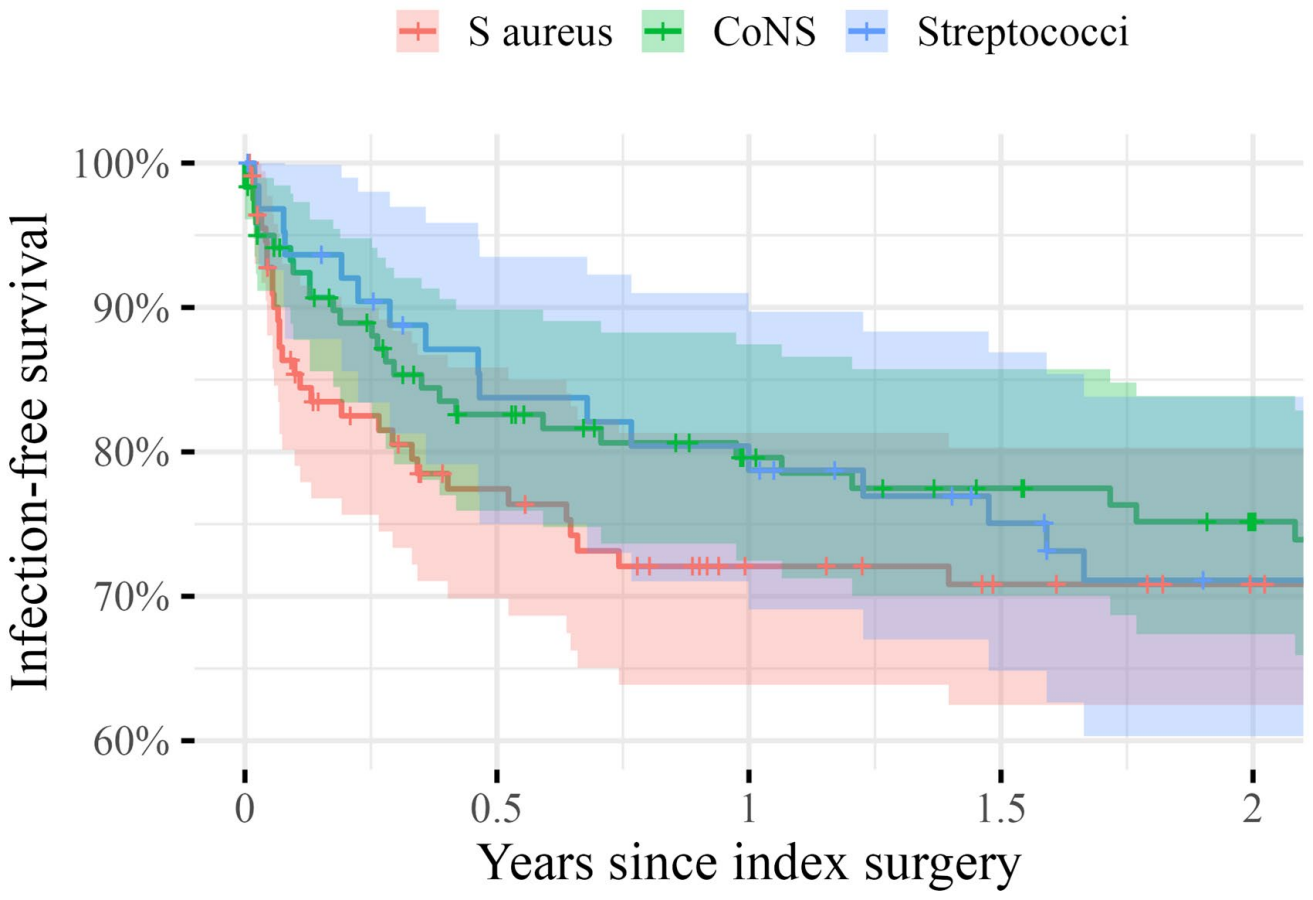
Unadjusted Kaplan–Meier survival curves giving the infection-free survival in percentages on the *y*-axis during the first 2 years after the index surgery (*x*-axis) for the three groups: *Staphylococci aurei* (*S. aureus*) in red, *Coagulase-negative Staphylococci* (*CoNS*) in green and *Streptococci* in blue. The shaded areas indicate 95% confidence intervals.

The unadjusted infection-free survival 2 years postoperatively was 71% (95%CI: 63–80) for *S. aureus*, 75% (95%CI: 67–84) for *CoNS*, and 60% (95%CI: 60–84) for *Streptococci* (Figure 2). After adjustment we found an insignificant HR for the risk of infection-relapse of 1.2 (95%CI: 0.7–2.0) for *CoNS* and 1.1 (95%CI: 0.6–2.0) for *Streptococci* with *S. aureus* serving as the reference.

**Figure 3:**
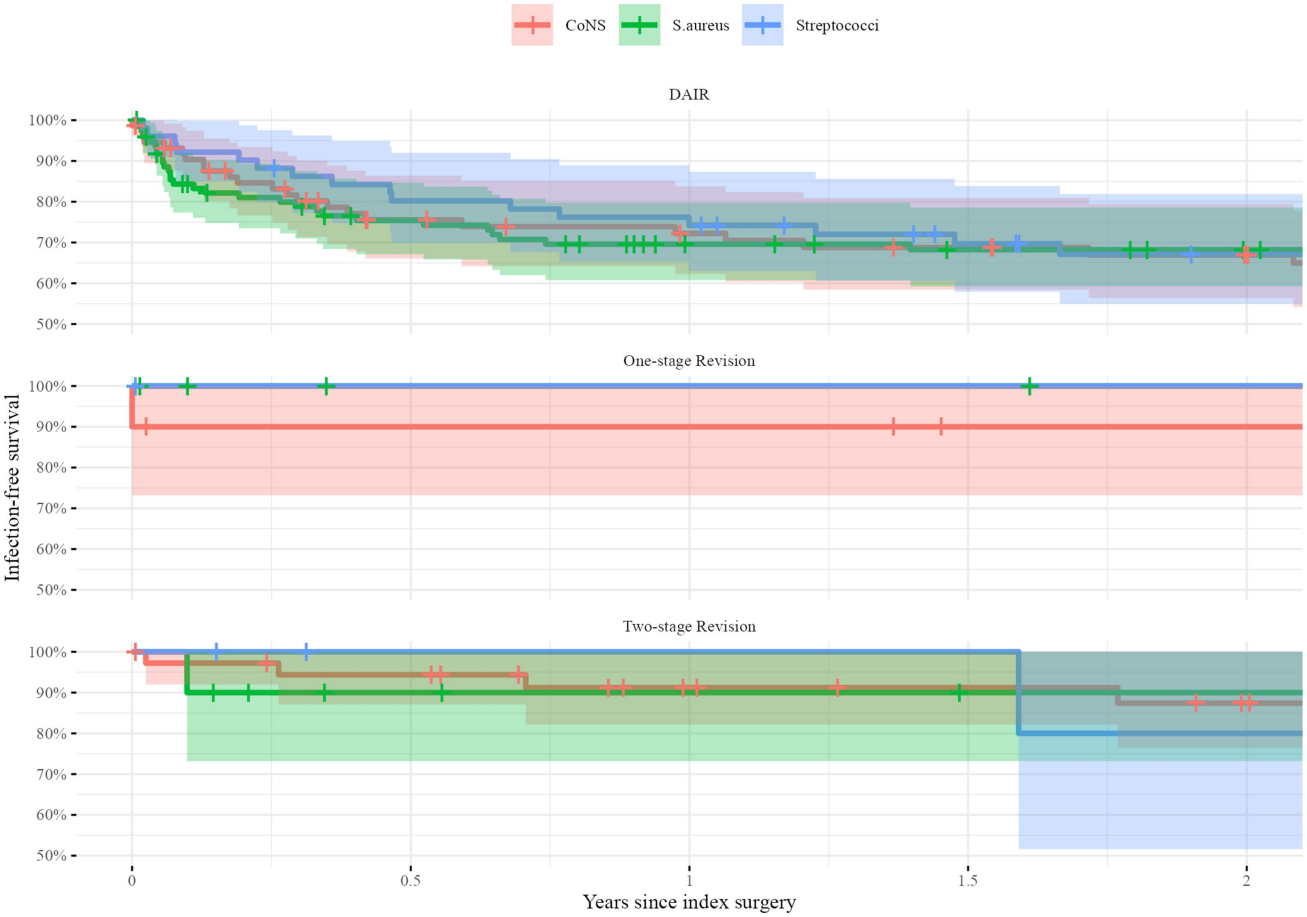
Unadjusted Kaplan–Meier survival curves stratified for the type of index surgery: Debridement, antibiotic and implant retention (DAIR, top), one-stage exchange (middle) and two-stage exchange (bottom). The infection-free survival in percentages is given on the *y*-axis during the first 2 years after the index surgery (*x*-axis) for the three groups: *Staphylococci aurei* (*S. aureus*) in red, *Coagulase-negative Staphylococci* (*CoNS*) in green and *Streptococci* in blue. The shaded areas indicate 95% confidence intervals.

In the stratified analyses, there were no differences in survival either. After DAIR, infection-free survival at 2 years was 68% (95%CI: 59–79) for *S. aureus*, 67% (95%CI: 56–79) for *CoNS*, and 67% (95%CI: 55–82) for *Streptococci*. Following one-stage revision, infection free-survival at 2 years was 100% for all pathogens. The infection-free survival 2 years after two-stage revision was 90% (95%CI: 73–100) for *S. aureus*, 87% (95%CI: 76–100) for *CoNS*, and 80% (95%CI: 52–100) for *Streptococci* (Figure 3).

## Discussion

We found similar infection-free survival rates between the investigated groups and no statistically significant increased risk of PJI relapse in patients affected by *CoNS* or *Streptococci* compared to *S. aureus*. Even when stratifying the analysis for the applied surgical method, we were unable to detect any differences in survival free from infection.

This stands in contrast to the findings of other authors reporting inferior results after PJI caused by *CoNS* or *Streptococci*. One study investigating the outcome of *CoNS*-caused knee PJI found that less than half of the PJI were treated successfully (Charalambous et al., 2022). However, that study defined treatment success in a different way and limited the analysis to the knee joint. Another study comparing the outcomes of *CoNS*-caused PJI with those caused by *S. aureus* after two-stage exchange did notice some differences in the way these entities present clinically, but failed to establish a difference in infection-free survival (Gao et al., 2019). Even after DAIR, no difference in survival free from infection after PJI caused by *S. aureus* or *CoNS* could be detected (Tornero et al., 2012).

Infection-free survival rate of patients affected with streptococcal PJI treated with DAIR in our study is congruent with several studies (Akgün et al., 2017; Fiaux et al., 2016; Lora-Tamayo et al., 2017), whereas others have indicated more successful relapse-free survival rate of patients affected by streptococcal PJI (Lam et al., 2018; Betz et al., 2015; Kherabi et al., 2022). Both streptococcal and staphylococcal PJI have a wide range of relapse-free survival rates when treated with DAIR. The reasons behind the varying relapse-free survival rates of PJI might be numerous: Some studies have defined the need for supplementary antibiotic treatment other than originally planned as a treatment failure (Lora-Tamayo et al., 2017), whereas others have not, raising the question of whether a standardized definition of treatment failure would grant a higher degree of comparability between different studies and surgical techniques. Furthermore, most studies within the field of PJI are underpowered to detect differences, as they by design often are smaller cohort studies. Lastly, the success of DAIR after PJI regardless of the underlying pathogen highly depends on the time span from disease manifestation and treatment initiation. Some studies do not report this paramount factor, while others do, but still might have suffer from a wide variety in their material. Our analysis of infection-free survival stratified for the onset of PJI indicated no differences between groups (Supplementary Figure S1).

One possible explanation for the larger proportion of treatment failure in PJI caused by *S. aureus* and *CoNS* compared to streptococcal PJI shown in other studies could be the increased prevalence of multi-drug-resistant strains, such as *MRSA*, in the staphylococcal spp., compared to the streptococcal spp. (Kherabi et al., 2022). This study included only one *MRSA* and one multi-drug-resistant *CoNS*. Studies excluding *MRSA* either completely or in certain analyses have also implicated a less favorable outcome of staphylococcal compared to streptococcal PJI, and suggest this is due to the staphylococcal ability to form biofilm (Espíndola et al., 2022). Of all staphylococcal PJI patients in this study, 33.6% were not treated with Rifampicin postoperatively. A recently published study indicates that the risk of treatment failure is increased fourfold in Rifampicin-resistant staphylococcal PJI compared to Rifampicin-sensitive staphylococcal PJI (Lazarinis et al., 2023).

### Limitations

This study had several limitations. First, the retrospective nature of this study limits the validity of our findings. Human sources of error due to retrospective examination of medical charts are apparent. Second, even though the final study population size consisted of a relatively large number of patients (*n* = 299), subgrouping by type of bacteria and surgical intervention rendered smaller subgroups not equal in size. The number of events compared to the total number of observations in this study was relatively low, resulting in relatively large confidence intervals representing considerable estimation uncertainty. Due to the scarcity of events, Cox regression models were deemed non-meaningful, for the stratified analysis. We chose to summarize all *CoNS* as one entity, but there is ample evidence that – among others – *S. lugdunensis*, while taxonomically a *CoNS*, clinically more resembles *S. aureus* (Lourtet-Hascoët et al., 2016; Herry et al., 2022). One major limitation to our study is the definition of both the exposure, PJI, and the outcome, relapse of PJI. Despite efforts in standardizing the diagnostic criteria, the diagnosis of PJI is dependent on a high degree of clinical expertise and correct interpretation of clinical and laboratory findings. In the most current attempt at categorizing the diagnosis of PJI the remaining level of uncertainty has been addressed by creating an intermediate level of “likely PJI.” We tried to follow the updated consensus definition (Shohat et al., 2019), but nonetheless a certain amount of uncertainty regarding the reliability of the PJI diagnosis of patients included in this study is present.

Another limitation to our study is the lack of clinical follow-up. To evaluate patient outcomes based only on the review of medical charts induces uncertainty as to whether patients suffered PJI relapses unknown to us. We believe this to be a limited problem since patients with relapses would mostly have been referred to our tertiary referral center if the need for renewed surgery had been apparent. Nonetheless, patients may have been considered too frail to undergo further surgery by their local physicians, or they may have declined further surgery, and opted for antibiotic suppressive treatment. However, we have no reason to believe that the probability of this scenario should be higher in one of the three investigated groups of patients, and we therefore believe that no considerable bias was introduced. Due to a large proportion of deceased or geographically very distant patients less than half of our study cohort would have been expected to attend clinical follow-up in any case. Outcomes were dated by date of renewed surgery due to infection, start of suppression therapy, date of amputation, death, or last medical chart entry from the orthopedic department before emigration. Possible reinfection once emigrated is lost due to loss to follow-up. This risk was minimized by censoring from the last date of medical entry before emigration, and only six patients emigrated, indicating that the uncertainty is small.

Lastly, whether the optimal surgical strategy was applied regarding time since onset of symptoms was not evaluated in this study. Thus, the question of whether patients received optimal surgical strategy, possibly influencing relapse-free survival rate in this study, remains unclear.

### Strengths

Our study also comes with some strengths. Most studies within the field of PJI are as mentioned earlier small in their sample size. This study included 299 PJI patients, and even the smallest sub-group of streptococcal PJI composed of 64 PJI. Thus, this is the largest retrospective cohort study comparing treatment outcomes after PJI caused by *S. aureus*, *CoNS* or *Streptococci*. Another strength is the stringent selection process that was used to delineate our study population: By excluding all polymicrobial infections, all patients who had PJI in another joint and all with incomplete data we attempted at reducing remaining uncertainties and dependency issues, rendering an observational study that to us appears as a valid description of surgically treated patients affected by monomicrobial PJI by the three included pathogens.

## Conclusion

As we found no difference in survival, we do not see the need to alter the surgical method applied based on the underlying pathogen. In other words, there is no need to be more aggressive. However, as surgical methods are only one pillar in the treatment of PJI, future studies should also consider the antibiotical treatment to illuminate how that affects the investigated outcomes.

## Data Availability

The datasets presented in this article are not readily available because due to the strict Swedish Law, data must not be shared with third parties. Limited, aggregated data can upon reasonable request be shared. Requests to access the datasets should be directed to anders.bruggemann@uu.se.
