# Supplementary material for: Never mind the bug: no differences in infection-free survival after periprosthetic joint infections with Staphylococcus aureus, Coagulase-negative Staphylococcus, or Streptococcus
Source: Front Microbiol. 2025 Jan 3;15:1503928. doi: 10.3389/fmicb.2024.1503928 (PMC11739087; doi:10.3389/fmicb.2024.1503928)
Supplement: Supplementary file 1 [file Table_1.docx]

| **Staphylococcal spp. intraoperative findings** | **Overall (n=235)** |  |
| --- | --- | --- |
|  |  |  |
| **CoNS** | **107 (45.6%)** |  |
| - *CoNS NFS* | *51 (21.7%)* |  |
| - *CoNS NFS (multi drug resistant)* | *1 (0.4%)* |  |
| - *S. capitis* | *6 (2.6%)* |  |
| - *S. caprae* | *2 (0.9%)* |  |
| - *S. epidermidis* | *42 (17.9%)* |  |
| - *S. lugdunensis* | *5 (2.1%)* |  |
| **S. aureus** | **100 (42.6%)** |  |
| - *S. aureus* | *99 (42.1%)* |  |
| - *MRSA* | *1 (0.4%)* |  |
| **No bacteria^a^** | **21 (8.9%)** |  |
| **No intraoperative biopsy^b^** | **6 (2.5%)** |  |
| **Streptococcal spp. intraoperative findings** | **Overall (n=64)** |  |
| **Alfa streptococcus group** | **15 (23.4%)** |  |
| - *Alfa streptococcus NFS* | *8 (12.5%)* |  |
| - *S. anginosus group* | *1 (1.6%)* |  |
| - *S. mitis group* | *2 (3.2%)* |  |
| - *S. salivaris group* | *1 (1.6%)* |  |
| - *S. sanguinis group* | *3 (4.7%)* |  |
| **Group B streptococcus** | **13 (20.3%)** |  |
| - *Group B Streptococcus NFS* | *1 (1.6%)* |  |
| - *S. agalactiae* | *12 (18.8%)* |  |
| **Group C Streptococcus NFS** | **3 (4.7%)** |  |
| **Group G streptococcus** | **19 (29.7%)** |  |
| - *Group G Streptococcus NFS* | *10 (15.6%)* |  |
| - *S. dysgalactiae* | *9 (14.1%)* |  |
| **S. pneumoniae** | **2 (3.1%)** |  |
| **S. pyogenes** | **2 (3.1%)** |  |
| **No bacteria^c^** | **8 (12.5%)** |  |
| **No intraoperative biopsy^d^** | **1 (1.6%)** |  |

*^a^= no intraoperative bacteria found but significant preoperative staphylococcal finding.*

*^b^= no intraoperative biopsy performed.*

*^c^= no intraoperative bacteria found but significant preoperative streptococcal finding.*

*^d^= no intraoperative biopsy performed.*

*NFS= not further specified*
