# Supplementary figures and images for: Never mind the bug: no differences in infection-free survival after periprosthetic joint infections with Staphylococcus aureus, Coagulase-negative Staphylococcus, or Streptococcus
Source: Front Microbiol. 2025 Jan 3;15:1503928. doi: 10.3389/fmicb.2024.1503928 (PMC11739087; doi:10.3389/fmicb.2024.1503928)

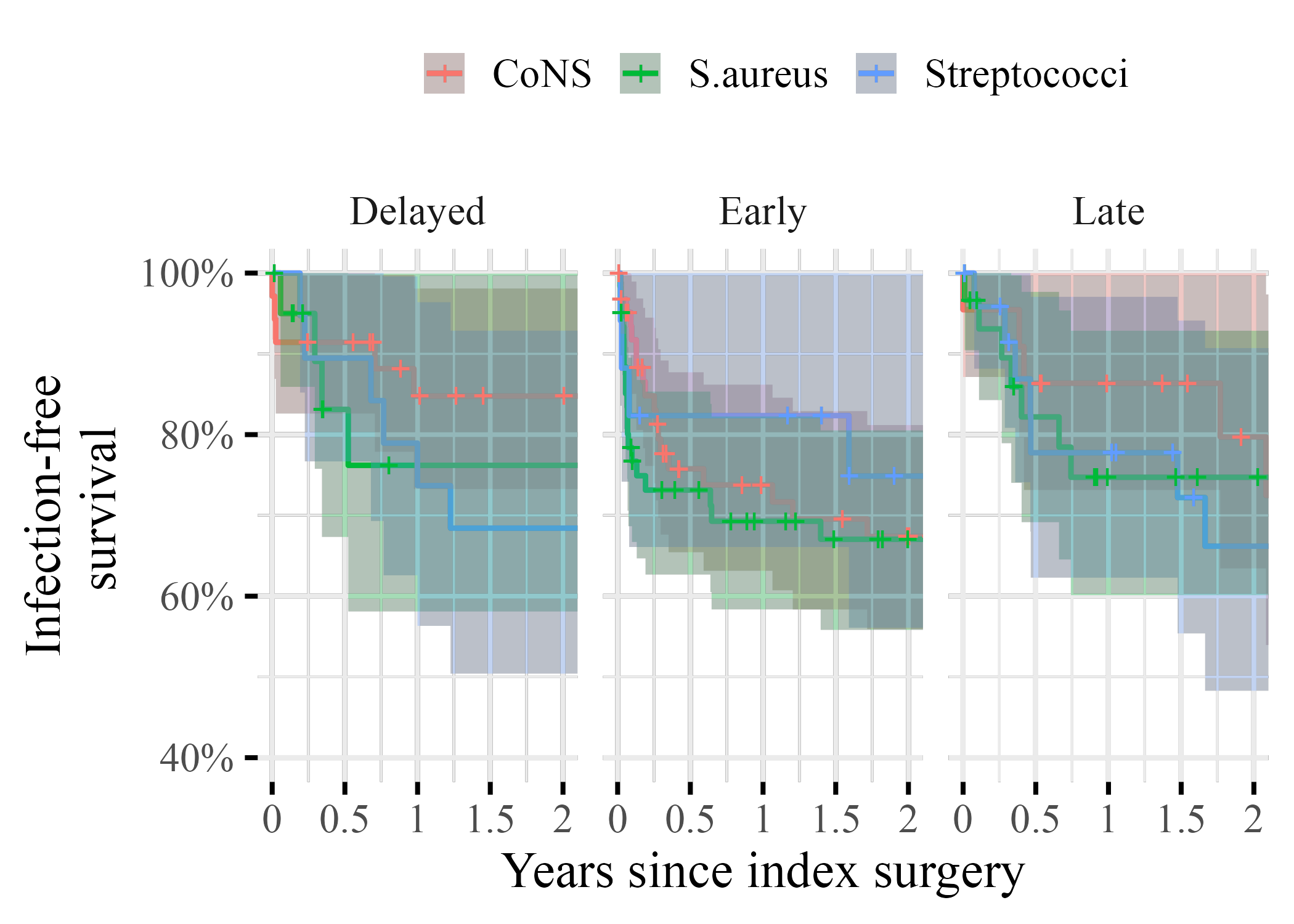

Supplement: Supplementary file 2 [file Image_1.tiff]
